# Optimization Research of Machining Parameters for Cutting GH4169 Based on Tool Vibration and Surface Roughness under High-Pressure Cooling

**DOI:** 10.3390/ma14247861

**Published:** 2021-12-18

**Authors:** Yali Zhang, Mingyang Wu, Keke Liu, Jianyu Zhang

**Affiliations:** Key Laboratory of Advanced Manufacturing and Intelligent Technology, Ministry of Education, Harbin University of Science and Technology, Harbin 150080, China; 1920100006@stu.hrbust.edu.cn (Y.Z.); 13136677353@163.com (K.L.); 1920100018@stu.hrbust.edu.cn (J.Z.)

**Keywords:** high-pressure cooling, tool vibration, surface roughness, support vector machine, NSGA-II genetic algorithm

## Abstract

The nickel-based superalloy is widely used in aerospace. It is a typical difficult-to-cut material with poor plasticity. During the cutting process, the fluctuation of the cutting force caused by the change of cutting conditions can aggravate tool vibration, thereby reducing the surface quality of the machined workpiece. However, the emergence of high-pressure cooling technology provides technical support for overcoming the difficulty in superalloy processing. Therefore, it is of great significance to optimize the tool vibration and surface roughness of cutting GH4169 under high-pressure cooling. Taking GH4169 as the research object, the single-factor and orthogonal high-pressure cooling cutting experiments were conducted firstly in this paper. Then, the methods of the main effect diagram and response surface were applied to analyze the impact of cutting speed, feed rate, cutting depth, and cooling pressure on the three-way tool vibration. Next, MATLAB was adopted to draw the frequency spectrum of radial tool vibration at different cutting speeds, and the relationship between chip morphology, tool vibration, and workpiece surface roughness at different cutting speeds was discussed. Based on this, a mathematical model of radial tool cutting vibration and surface roughness related to the cutting amount and cooling pressure was established. Support vector machine (SVM) was applied to make predictions. Meanwhile, the non-dominated sorting genetic algorithm with an elitist strategy was adopted for multi-objective optimization, and the optimization results were verified through experiments. The results indicated that the feed rate and cutting depth had a great impact on the tool vibration and surface roughness. The established mathematical model was accurate and effective for optimizing the cutting parameters. These results are of great significance to improve the cutting stability and the quality of machined surface.

## 1. Introduction

The nickel-based superalloy is widely used in the manufacture of aero-engine turbine disks. It is because of its excellent mechanical properties and corrosion resistance at high temperatures. GH4169 is the most extensively used nickel-based superalloy material in aero-engine history [1,2]. GH4169 is a typical difficult-to-cut material with high strength and low thermal conductivity. During the cutting process, it is easy to have such phenomena, such as large cutting force, high cutting temperature, and difficult chip breaking [3,4]. In addition, the fluctuation of the cutting force caused by the change of cutting conditions can aggravate the vibration of the workpiece-tool system, thereby causing cutting vibration. This type of vibration can change the relative position between the tool and the workpiece, which will lead to changes in the cutting depth of the tool and then make the machined surface uneven. As a result, the surface roughness will be increased [5]. Tool vibration can also lead to the premature fatigue failure of machine tool parts. In this case, the reliability, safety, and strength of the machine tool decrease, reducing the efficiency of cutting processing. High-pressure cooling is a new type of cooling and lubrication auxiliary processing technology in high-efficiency metal cutting. It can effectively improve the machinability and surface quality of difficult-to-cut materials. Therefore, it is necessary to optimize the machining parameters based on the tool vibration and surface roughness of cutting GH4169 under high-pressure cooling.

Currently, aiming at the cutting problems of difficult-to-cut materials, considerable efforts have been devoted to the research on cutting vibration and surface roughness. Zhao et al. [6] conducted a low-frequency vibration drilling test on titanium alloy TC4 by a single-factor and orthogonal test method. They analyzed the influence of drilling parameters and vibration parameters on the drilling force. The results showed that the chip breaking was reliable and the chip removal was smooth when the ratio of amplitude to feed rate was close to the critical chip-breaking value of 0.81. Grzesik et al. [7] used the size of the power spectrum to determine the effect of cutting vibration on the surface profile under variable feed rates. Zhao et al. [8] conducted experiments on the high-speed milling of slender structural parts. In addition, they established an optimization model with machining efficiency and tool life as the objective function and cutting parameters as variables. Based on the model, the global optimal solution of milling parameters was obtained through particle swarm optimization. Hüseyin et al. [9] used dry cutting and minimum quantity lubrication for turning AISI4140 steel and analyzed the experimental results through the signal-to-noise ratio. They found that compared to dry machining, minimum quantity lubrication had a greater impact on the cutting force and surface roughness. Li et al. [10] analyzed the influence of various process parameters on the material removal rate, tool life, and residual stress layer thickness. Based on the analysis, they determined the optimization plan of process parameters for titanium alloy disc milling and slotting. Hong-seok et al. [11] maximized the cutting force by optimizing the feed rate, thereby reducing the processing time and improving the efficiency. Kong et al. [12] exploited the orthogonal experiment method to perform the titanium alloy micro-lubrication system, thus determining the optimal system parameter combination. Based on the results of the hardened steel precision turning test, Wang [13] established an amplitude prediction model of tool vibration using the BP neural network algorithm. Then, using the established model, comprehensive optimization was performed on the cutting parameters of the machining process to obtain the optimal cutting combination. Ratnam et al. [14] studied the tool vibration in the process of turning and milling extruded brass through orthogonal experiments. They found that the tool vibration increased with the decrease in the spindle speed, leading to tool wear and a gradual increase in tool cost. Based on the high temperature and low cycle fatigue test of turning superalloy Inconel 718, Ren et al. [15] analyzed the sensitivity of turning process parameters to high temperature and low cycle fatigue life, and they optimized the process parameters with anti-fatigue life as the goal. Through experimental observation, Krolczyk et al. [16] found that power spectrum density analysis enabled determining the amplitude of the feed component, which was observed for higher ranges of feed. For low feed values, cutting tool vibrations and tool edge wear were dominant on surfaces. Podulka et al. [17] used power spectral density software to study the influence of reducing high-frequency noise on surface topography measurement results. Sharma et al. [18] found in their study that after wavelet filtering of reconstructed images with a low signal-to-noise ratio, the obtained image’s signal-to-noise ratio is larger than the image composition.

In view of the above analysis, in this paper, single-factor and orthogonal experimental methods were exploited to conduct experiments on cutting GH4169 under high-pressure cooling conditions. Meanwhile, the main effect diagram and response surface method were adopted to analyze the influence of cutting amount and cooling pressure on the tool cutting vibration and surface roughness. Through the regress tool provided by the MATLAB toolbox, a mathematical model was established with cutting amount and cooling pressure as independent variables, and radial tool vibration acceleration and surface roughness as dependent variables. A support vector machine (SVM) was used to predict the results, and the NSGA-II genetic algorithm was used to establish a mathematical optimization model with radial tool vibration acceleration, surface roughness, and cutting efficiency as the optimization objectives. Moreover, the optimization results were verified through experiments. The optimization results provide an important theoretical basis for the selection of cutting parameters in actual machining under high-pressure cooling.

## 2. Cutting Vibration Theory and Dynamic Model

### 2.1. Dynamic Model Structure of Turning Vibration System

**Figure 1:**
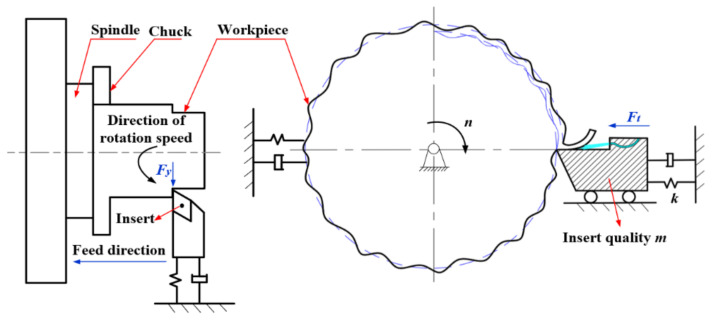
The dynamic model of turning vibration system.

During the cutting process, the tool vibration mainly includes self-excited vibration, forced vibration, and free vibration. In this paper, the GH4169 superalloy turning experiment under high-pressure cooling belonged to the continuous cutting. The high-pressure coolant mainly played a lubricating effect on the cutting vibration, i.e., damping coefficient. At this time, the tool could be regarded as an elastic system, and the workpiece was a rigid body relative to the tool. The two interacted with each other to generate vibration. The dynamic model of this turning vibration system can be simplified to a single degree of freedom regenerative self-excited vibration system model, as shown in Figure 1.

### 2.2. Data Processing of Vibration Signal

#### 2.2.1. Establishment of the Finite Element Simulation Model of Milling Process

In the actual cutting process, the collected vibration signals are mixed with external noise signals and contain interference frequencies. This can cause distortion, amplitude fading, and phase deviation, leading to the increase in error rate and then signal distortion [19,20]. Thus, the collected original vibration signals were first preprocessed by wavelet denoising. The model of the signals mixed with external noise is shown in Formula (1):

(1) $$s\left(k\right)=x\left(k\right)+\varepsilon \cdot e\left(k\right),\;k=0,1,\ldots ,n-1$$

where *s*(*k*) is the noise-contained signal mixed in the signal; *x*(*k*)is the measured useful signal; *ε* is the noise intensity; and *e*(*k*) is the noise signal.

In this paper, according to the wavelet denoising algorithm program [21,22], the wavelet denoising preprocessing of the tool vibration acceleration signal is carried out by using MATLAB software (R2018a, MathWorks, Natick, MA, USA) programming. Some seriously deviated signals and interference signals such as high-frequency random noise are removed from the signal, and only the effective tool vibration acceleration signal is retained.

**Figure 2:**
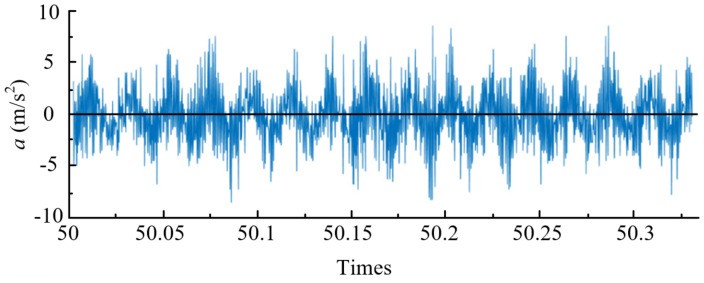
Time domain diagram of vibration acceleration of original tool (*v* = 75 m/min, *f* = 0.05 mm/r, *a_p_* = 0.4 mm, *P* = 50 bar).

**Figure 3:**
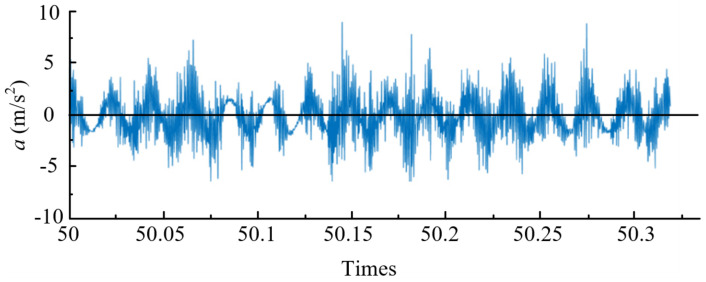
Time domain diagram of tool vibration characteristics after noise reduction and filtering (*v* = 125 m/min, *f* = 0.05 mm/r, *a_p_* = 0.4 mm, *P* = 50 bar).

Figure 2 is the original vibration signal before noise reduction, and Figure 3 is the vibration acceleration signal after wavelet noise reduction. By comparing the results of the two figures, it can be found that the clutter signal is significantly reduced, and the effect of wavelet noise reduction signal is more significant.

#### 2.2.2. RMS Value of Vibration Signal

There are errors in the vibration signals detected by the experiment. When the signal is analyzed in the time domain, the root-mean-square (RMS) method can make the errors smaller. RMS is an effective parameter for representing the signal strength, and it is also referred to as the effective value of acceleration. It can reflect the stability of the entire cutting process [23]. The calculation formula of RMS is shown in Formula (2):

(2) $$\mathrm{RMS}=\sqrt{\frac{\sum_{i=1}^{N}X_{i}^{2}}{N}}$$

where *x_i_* denotes the sampling signal, and *N* denotes the sampling point.

#### 2.2.3. Time-Frequency Domain Analysis of Vibration Signals

The time-domain analysis method records signals through time, and it is very simple and intuitive. Hence, the amplitude of the vibration can be obtained by this method. The frequency-domain analysis performs fast Fourier transform (FFT) on the collected time-domain vibration signals. The formula of Fourier transform is shown in Formula (3):

(3) $$x\left(w\right)=\int_{R}x\left(t\right)e^{-jwt}dt.$$

Based on the FFT, the complex vibration signals were decomposed into various vibration frequencies, and the frequency spectrum of the vibration signals was obtained. In this way, the vibration amplitude of the frequency can be analyzed to obtain ideal information and data.

## 3. Experimental Study on Cutting GH4169 Superalloy under High-Pressure Cooling

During the cutting process, vibration generally occurs, and the cutting force also causes cutting vibration. The vibrations due to different factors affect each other and finally all act on the tool to form tool vibration. Meanwhile, the vibration can change the relative position between the tool and the workpiece, which in turn causes changes in the cutting depth of the tool and finally leads to the uneven machined surface. The surface roughness is an important characterization parameter of surface morphology, which affects the accuracy, fatigue strength, contact stiffness, and wear resistance of the workpiece [24]. To achieve the optimal cutting performance and ensure the quality of the machined surface, single-factor and orthogonal experiments were used to study the influence of different cutting conditions on tool vibration and surface roughness.

### 3.1. Experiment Conditions

**Figure 4:**
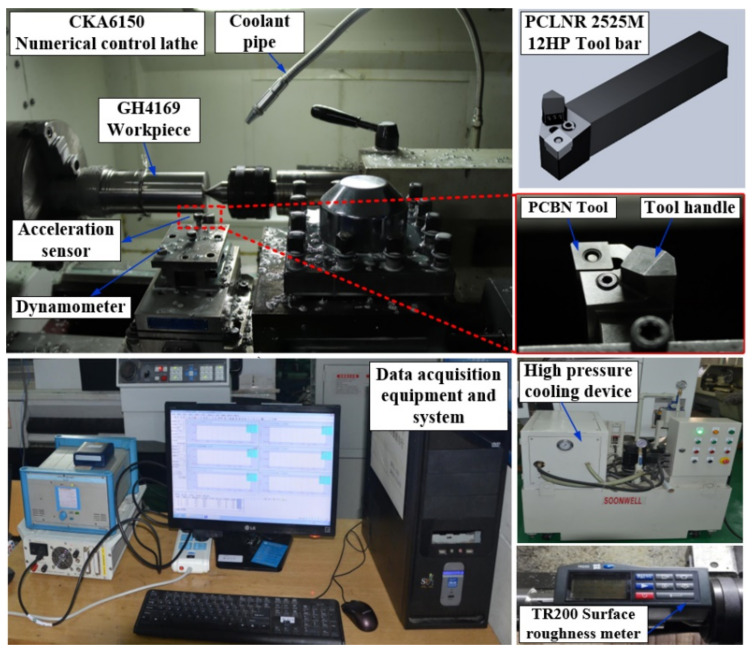
Turning experiment device.

The turning experiment device used in this study is shown in Figure 4. The CNC machine tool is CAK6150 (Dalian, China), and the workpiece is a cylindrical bar of nickel-based superalloy GH4169 with Φ65 × 400 mm. The main chemical composition of the workpiece is listed in Table 1. The tool is the CNGA120408-2 indexable diamond PCBN tool manufactured by Zhuzhou Diamond Cutting Tool Company (Zhuzhou, China) with a rake angle and inclination angle of −6°, a relief angle of 6°, and a tool cutting edge angle of 95°. The PCLNR-2525M12HP Sandvik high-pressure cooling tool holder (Sandviken, Sweden) was also used.

**Figure 5:**
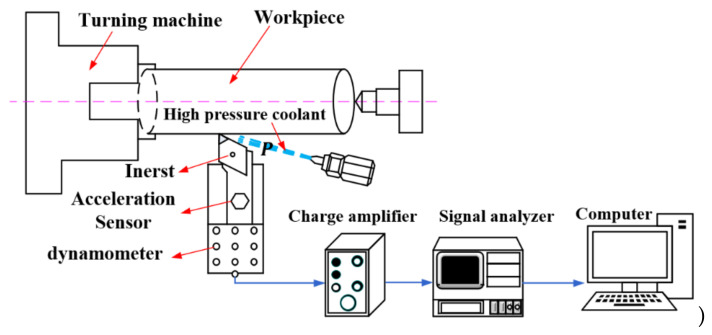
Experimental measurement system.

The measurement system for the turning experiment was mainly composed of machine tools, cutting tools, workpieces, and vibration signal collection systems. The tool vibration signals were collected by the Donghua DH5922 measurement system (Zhuzhou, China) at a sampling frequency of 5000 Hz. Meanwhile, the PCB acceleration sensor (356A02, PCB PIEZOTRONICS, Depew, NY, USA) with a sensitivity of 10.42 mV/g was used to obtain the vibration acceleration in three directions of the tool. The obtained vibration acceleration was input into the computer through the data acquisition card for data collection and analysis. The principle of the measurement system is shown in Figure 5. The machined surface roughness was measured by the handheld surface roughness meter TR200 (Times, Beijing, China) illustrated in Figure 4. The resolution was 0.01 μm and the sampling length was 0.8 mm. In order to reduce the test error, the machined surface roughness was obtained by measuring the average value at three different locations under each parameter, and the surface roughness *R_a_* was recorded.

### 3.2. Experiment Method

The experiments in this paper were divided into two parts: (1) single-factor experiments and (2) orthogonal experiments. The single-factor experiments were conducted to determine the influence of cutting amount and cooling pressure on tool vibration. The orthogonal experiments were conducted to find out the influence of the interaction between different cutting amounts and cooling pressure on tool vibration and surface roughness.

#### 3.2.1. Single-Factor Experimental Scheme

Single-factor experiments were performed to study the impact of different cutting amounts and cooling pressure on the tool vibration. To avoid the influence of tool wear on the experimental results, after the cutting in each set of experiments was completed, the tool was replaced with a new one in time. The feed rate has a greater impact on the tool vibration and surface roughness, and it has a large variation range. Hence, to find the impact closer to the actual situation, a total of 20 sets of experiments were conducted based on the same cutting amount and cooling pressure (*v*_c_ = 125 m/min, *f* = 0.05 mm/r, *a_p_* = 0.4 mm, *P* = 50 bar). The specific parameters used in the experiments were listed in Table 2.

As for groups 1–5, the feed rate, cutting depth, and cooling pressure remained unchanged, while the cutting speed gradually increased; as for groups 6–10, the cutting speed, cutting depth, and cooling pressure remained unchanged, while the feed rate gradually increased; as for groups 11–15, the cutting speed, feed rate, and cooling pressure remained unchanged, while the cutting depth gradually increased; as for groups 16–20, the cutting speed, feed rate, and cutting depth remained unchanged, while the cooling pressure gradually increased.

#### 3.2.2. Orthogonal Experimental Scheme

To obtain the optimal cutting combination and achieve a high-quality cutting, orthogonal experiments of vibration and surface roughness of cutting GH4169 tools under high-pressure cooling were designed based on the single-factor experiments. The orthogonal experiment method is to arrange the experiment reasonably through the orthogonal table and use as few experiment groups as possible to determine the influence of each factor on the experimental results. This method can consider the influence of the interaction between the factors on the experimental results. In these experiments, the cutting speed, feed rate, cutting depth, and cooling pressure were used as influencing factors, while the tool vibration and surface roughness were used as evaluation indexes. According to the principle of metal cutting and considering the actual production, the value ranges of the cutting speed, feed rate, cutting depth, and cooling pressure were 55–160 m/min, 0.05–0.14 mm/r, 0.3–0.6 mm, and 20–110 bar, respectively. The specific orthogonal experiment factors and level combinations are listed in Table 3.

### 3.3. Discussion of Experimental Results

During the cutting process, the tool vibration and surface roughness can be affected by a large number of factors, including machine tool accuracy, tool and workpiece materials, tool geometric parameters, cutting conditions, and cutting environment. Among them, machine tools, cutting tools, and workpiece materials are fixed factors, while cutting conditions are variable factors [25]. The cutting conditions mainly include cutting speed, feed rate, cutting depth, and cooling pressure. To achieve optimal cutting performance and ensure the quality of the machined surface, this paper mainly studied the impact of cutting conditions on tool vibration.

#### 3.3.1. The Influence Law of Cutting Speed on Tool Vibration

**Figure 6:**
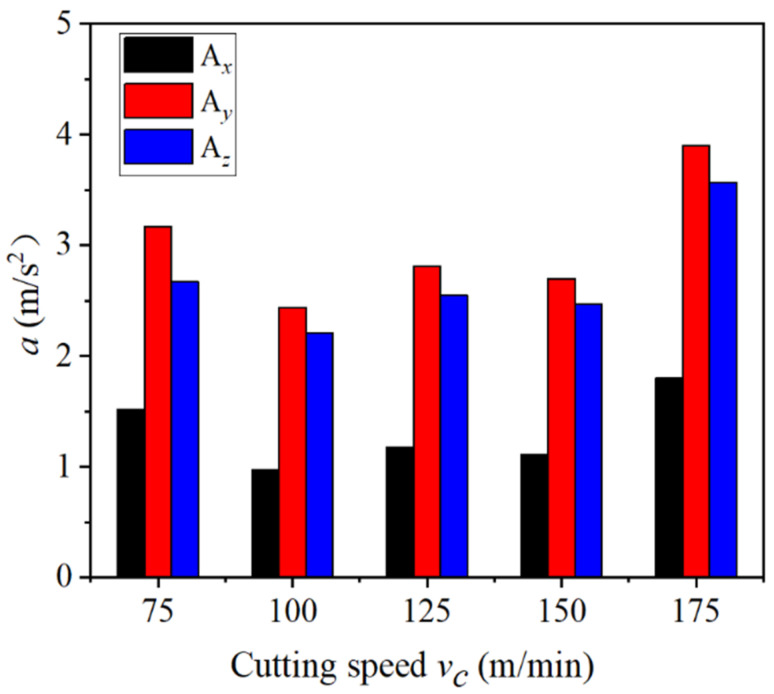
Influence of different cutting speeds on tool vibration.

**Figure 7:**
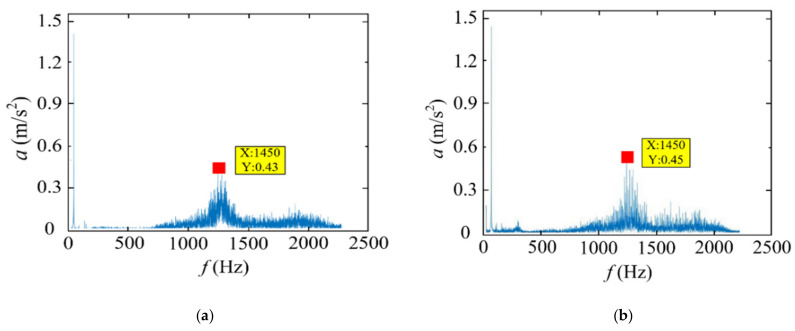

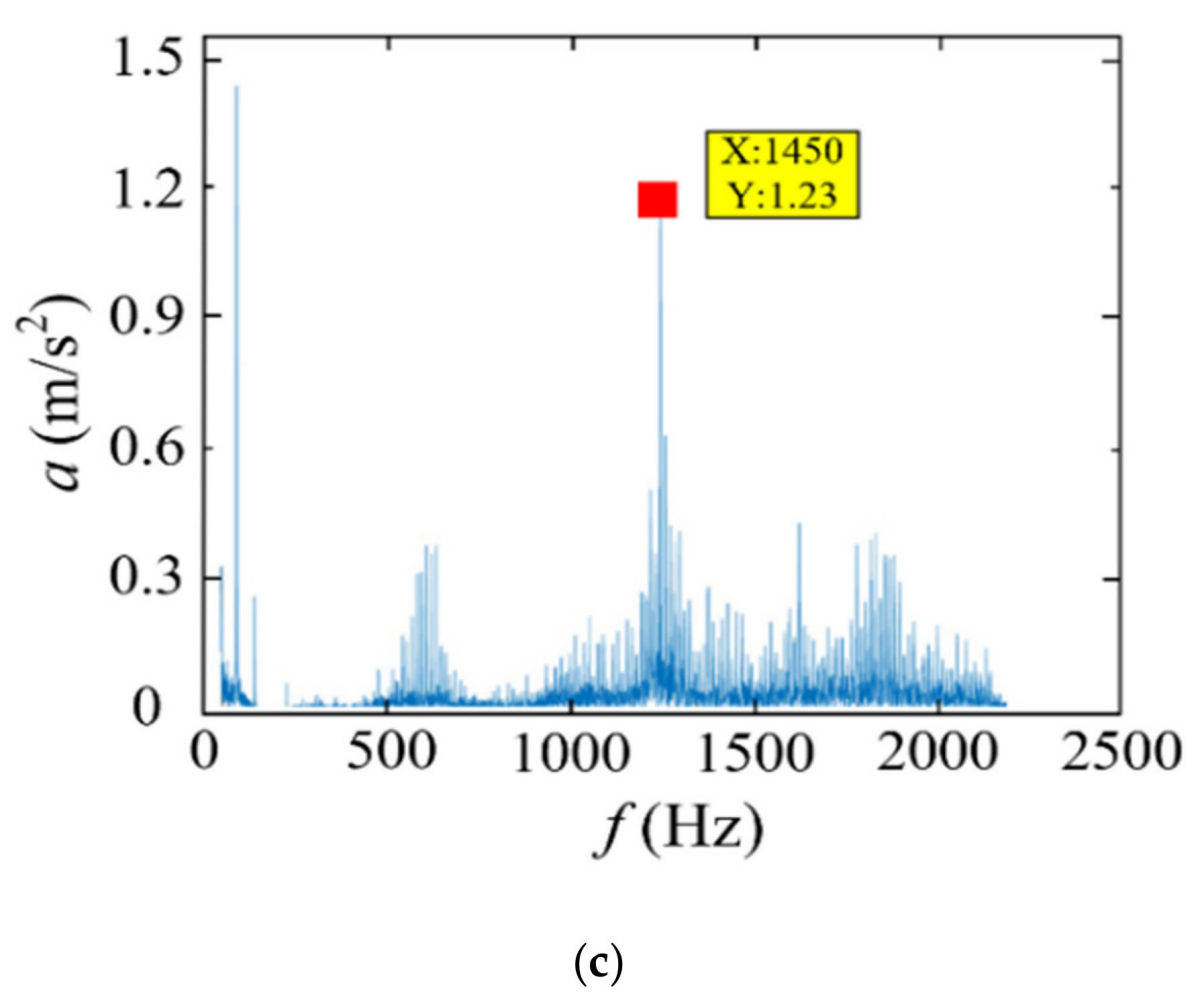
Spectrum diagram of radial tool vibration acceleration changing with cutting speed (*f* = 0.05 mm/r, *a_p_* = 0.05 mm, *P* = 50 bar): (**a**) *v_c_* = 75 m/min; (**b**) *v_c_* = 125 m/min; (**c**) *v_c_* = 175 m/min.

Figure 6 illustrates the influence of different cutting speeds on tool vibration acceleration. When the cutting speed was in the range of 75–175 m/min, the vibration acceleration of the three-way tool firstly decreased; then, they stabilized and finally increased gradually; when the cutting speed was in the range of 75–100 m/min and 125–150 m/min, the vibration acceleration decreased slowly; when the cutting speed was in the range of 100–125 m/min and 150–175 m/min, the vibration acceleration increased slowly; when the cutting speed was at 100 m/min, the vibration acceleration of the three-way tool was minimal. According to the principle of metal cutting, cutting speed has a great correlation with the tool vibration. As for the three-way vibrations of the tool, the radial tool vibration had the largest acceleration and the most obvious amplitude of variation. Therefore, the radial tool vibration acceleration was taken as the research object. The tool vibration signals collected by the measurement system were first preprocessed according to Formula (1), including elimination and wavelet noise reduction. Then, the preprocessed data were imported into MATLAB. The Fourier formula calculation and programming were performed according to Formula (3) to obtain the frequency spectrum of the radial tool vibration acceleration at different cutting speeds in Figure 7. It can be seen from Figure 7 that when the cutting speed is in the range of 75–175 m/min, the main vibration frequency of the tool was about 1450 Hz, that is, the frequency corresponding to the maximum acceleration position. With the increase in the cutting speed, the peak acceleration of the main vibration frequency of the tool gradually increases. When the cutting speed of 75 m/min, the peak acceleration of the main vibration frequency of the tool was 0.43 m/s^2^, and when the cutting speed was 175 m/min, the peak acceleration of the tool’s main vibration frequency increases to 1.23 m/s^2^.

Combined with Figure 6 analysis, this is mainly because when the cutting speed is 75–100 m/min, with the increase in cutting speed and the influence of adiabatic shear, tool vibration acceleration decreases. Meanwhile, when the cutting speed was more than 100 m/min, the temperature of the cutting area gradually rose, resulting in the degree of plastic deformation of the machined surface increasing, and with the continuous increase in the cutting speed, the friction between the tool and the workpiece is intensified. These two reasons lead to the increase in the resistance of the tool when cutting. At this time, the shape of the chip was serrated. The serrated chip makes the tool produce high-frequency vibration, which increases the vibration acceleration of the tool. The cutting speed was correlated with the vibration of the tool.

#### 3.3.2. The Influence Law of Feed Rate on Tool Vibration

**Figure 8:**
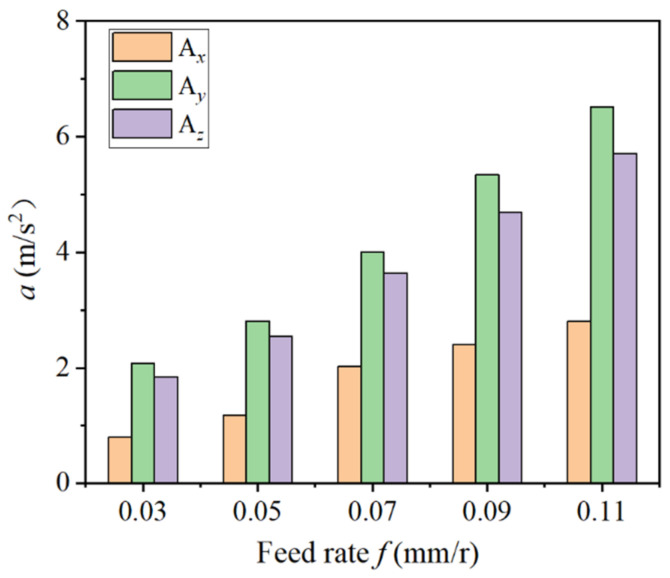
Influence of different feed rates on tool vibrational.

Figure 8 illustrates the influence of different feed rates on tool vibration acceleration. It can be seen that when the feed rate was within the range of 0.03–0.11 mm/r, the vibration acceleration of the three-way tool all displayed an upward trend. In addition, the feed rate had the greatest impact on the radial tool cutting vibration. The vibration acceleration of the tool increased with the feed rate. After the feed rate reached 0.05 mm/r, the increasing trend of vibration acceleration amplitude was more obvious.

#### 3.3.3. The Influence Law of Cutting Depth on Tool Vibration

**Figure 9:**
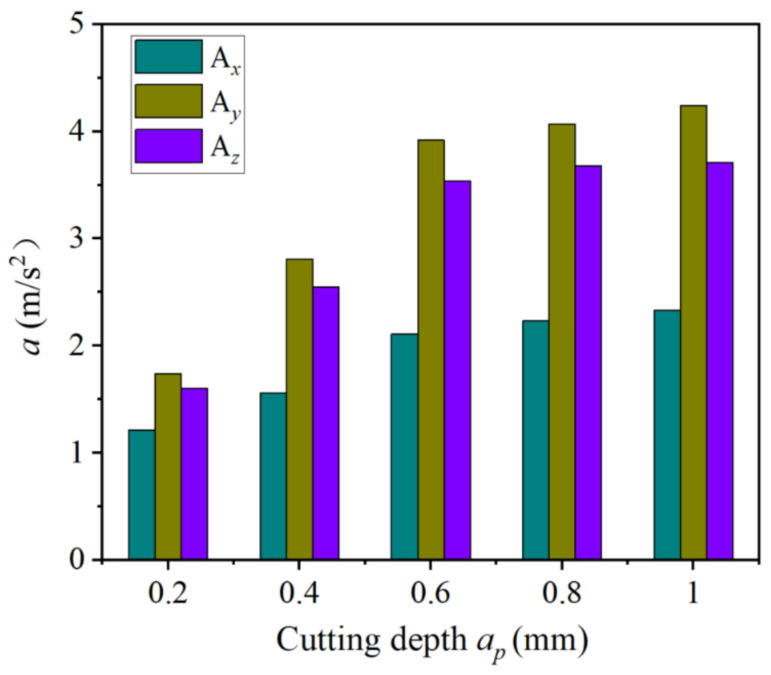
Influence of different cutting depths on tool vibrational.

Figure 9 illustrates the influence of different cutting depths on tool vibration acceleration. It can be seen that when the cutting depth was in the range of 0.2–1.0 mm, the change of the vibration acceleration of the three-way tool displayed an upward trend. Meanwhile, the cutting depth had the greatest impact on the radial tool cutting vibration. The tool vibration acceleration increased rapidly when the cutting depth was between 0.2 and 0.6 mm, and it decreased slightly when the cutting depth was in the range of 0.6–1.0 mm.

#### 3.3.4. The Influence Law of Cooling Pressure on Tool Vibration

**Figure 10:**
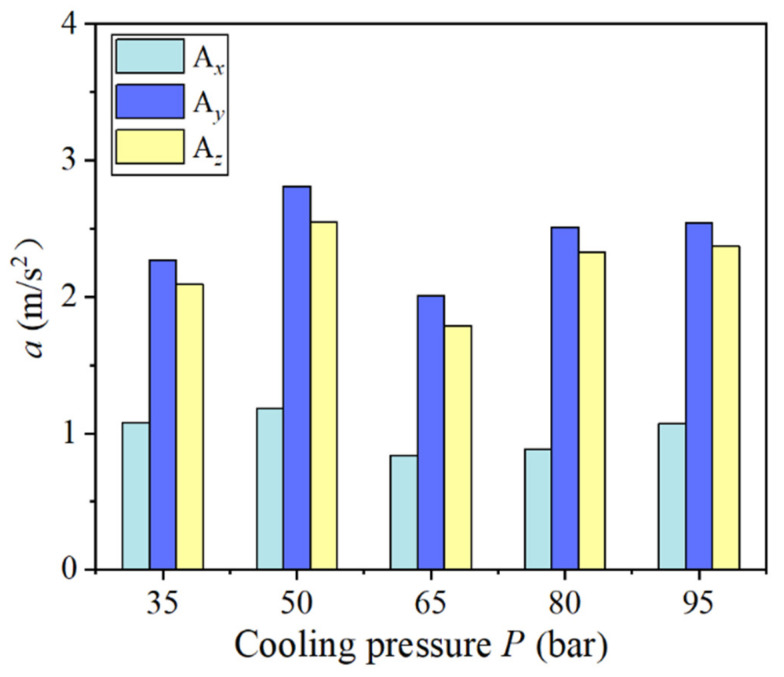
Influence of different cooling pressures on tool vibration.

Figure 10 illustrates the influence of different cooling pressures on tool vibration acceleration. It can be seen that when the cooling pressure was within the range of 35–95 bar, as the cooling pressure increased, the overall vibration acceleration value of the three-way tool changed little. In comparison, the cooling pressure had the greatest impact on the radial tool cutting vibration. When the cooling pressure was 65 bar, the cutting vibration acceleration was the lowest. This is mainly because the cutting force did not change suddenly with the increase in cooling pressure under the same cutting amount. Hence, the amplitude of tool vibration acceleration did not change significantly with the increase in cooling pressure.

In summary, the feed rate caused a greater change of the tool vibration than the cutting speed, cutting depth, and cooling pressure. Therefore, the feed rate had the most significant impact on tool vibration. This is because the increase in the feed rate increased the thickness of the cutting layer and the residual height of the surface roughness. In addition, as the cutting force increased, the cutting vibration increased, resulting in an increase in the surface roughness. However, a larger feed rate is not always better. When the feed rate reaches a certain value, even if the cutting force increases significantly, the tool vibration acceleration and surface roughness values will not increase significantly. Thus, appropriate cutting parameters should be selected in the process of cutting superalloy. However, the single-factor experiment design still has some shortcomings. It does not consider the interaction between factors but defaults to no correlation [26]. Therefore, the optimal cutting combination cannot be obtained, and it needs to be confirmed through an orthogonal experiment.

### 3.4. Orthogonal Experimental Results and Analysis

Based on the result analysis of the single-factor experiments, the orthogonal experiments were performed following the experimental scheme arranged in Table 3. A total of 16 experiments were conducted. The obtained results of the orthogonal experiments are listed in Table 4.

#### 3.4.1. Main Effect Diagram Analysis

**Figure 11:**
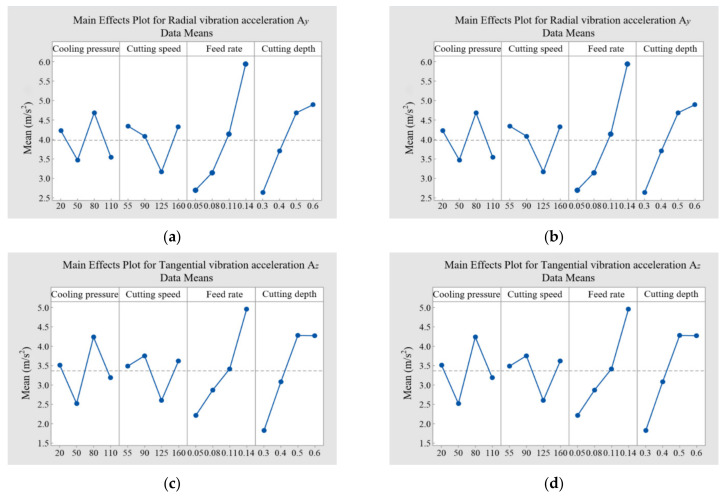
Main effect diagram of tool vibration and surface roughness under different processing parameters: (**a**) tool axial vibration; (**b**) tool radial vibration; (**c**) cutter tangential vibration; (**d**) surface roughness.

The main effects diagram can display the influence of multiple factors on the response at the same time, and it can compare the results of input parameters at different stages in a graphical manner. The horizontal line in the diagram represents the average of all response values in the experimental data table. The closer the point to the average line, the smaller its influence on the response parameters. The point above or below the average line has a greater influence on the response parameters, and its effect is reflected by the difference between the average value and the graph line. In this study, Minitab software (Minitab 19, State College, PA, USA) was exploited to analyze the main effects of cutting amount and cooling pressure on tool vibration and surface roughness, as shown in Figure 11.

As shown in Figure 11a–c, the cutting amount and cooling pressure had the same order of impacts on the three directions of the tool vibration, which is consistent with the results of the single-factor analysis. The order of impacts from large to small is feed rate, cutting depth, cooling pressure, and cutting speed. When the cutting speed was 125 m/min, the feed rate was 0.05 mm/r, the cutting depth was 0.3 mm, the cooling pressure was 50 bar, and the radial and tangential tool vibration acceleration values were minimal. When the cutting speed was 160 m/min, the feed rate was 0.05 mm/r, the cutting depth was 0.4 mm, the cooling pressure was 20 bar, and the axial tool vibration acceleration was minimal.

**Figure 12:**
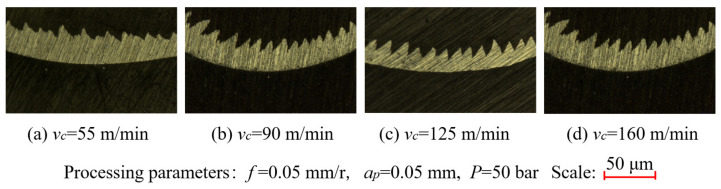
Morphology of the serrated chip under different cutting speeds (× 300): (**a**) cutting speed of 55 m/min; (**b**) cutting speed of 90 m/min; (**c**) cutting speed of 125 m/min; (**d**) cutting speed of 160 m/min.

As shown in Figure 11d, when the cooling pressure was in the ranges of 20–50 bar and 80–110 bar, the machined surface roughness decreased gradually; when the cooling pressure was between 50 and 80 bar, the surface roughness increased. However, the overall change amplitude of the surface roughness was small, indicating that the cooling pressure had little influence on the surface roughness. The surface roughness gradually decreased when the cutting speed was in the range of 55–125 m/min, and it increased suddenly when the cutting speed exceeded 125 m/min. This is mainly because the cutting temperature increases with the cutting speed, which leads to the occurrence of metal softening, the reduction of cutting vibration, and the decrease in surface roughness. However, the thermal conductivity of the GH4169 workpiece is low. The further increase in the cutting speed can result in a large accumulation of the cutting heat near the shear zone. In this case, the cutting heat cannot diffuse in a short time. Meanwhile, the resulting thermal softening effect is enhanced, which leads to the instability of metal adiabatic shear and increases the sawtooth degree of the chip. In addition, the fluctuation of the cutting force generated by the chip sawtooth aggravates the tool vibration, increasing the surface roughness of the machined surface. A VHX-1000 ultra-depth-of-field microscope (Keyence, Osaka, Japan) was used to observe the sawtooth chip morphology at different cutting speeds, and the observation results are shown in Figure 12. When the feed rate was between 0.05 and 0.14 mm, the surface roughness increased. This is mainly because as the feed rate increases, the cutting area of the tool also increases. In addition, the friction between the tool and the workpiece increases, which causes the tool cutting vibration to increase and leads to an increase in the surface roughness. When the cutting depth was in the range of 0.3–0.6 mm, as the cutting depth increased, the overall change amplitude of the surface roughness was little, indicating that the cutting depth had little influence on the surface roughness. The most significant factor affecting the surface roughness is the feed rate, which is followed by the cutting speed, the cutting depth, and the cooling pressure. Thus, the minimum surface roughness can be achieved with the cutting speed of 125 m/min, the feed rate of 0.05 mm/r, the cutting depth of 0.4 mm, and the cooling pressure of 50 bar.

#### 3.4.2. Response Surface Analysis

**Figure 13:**
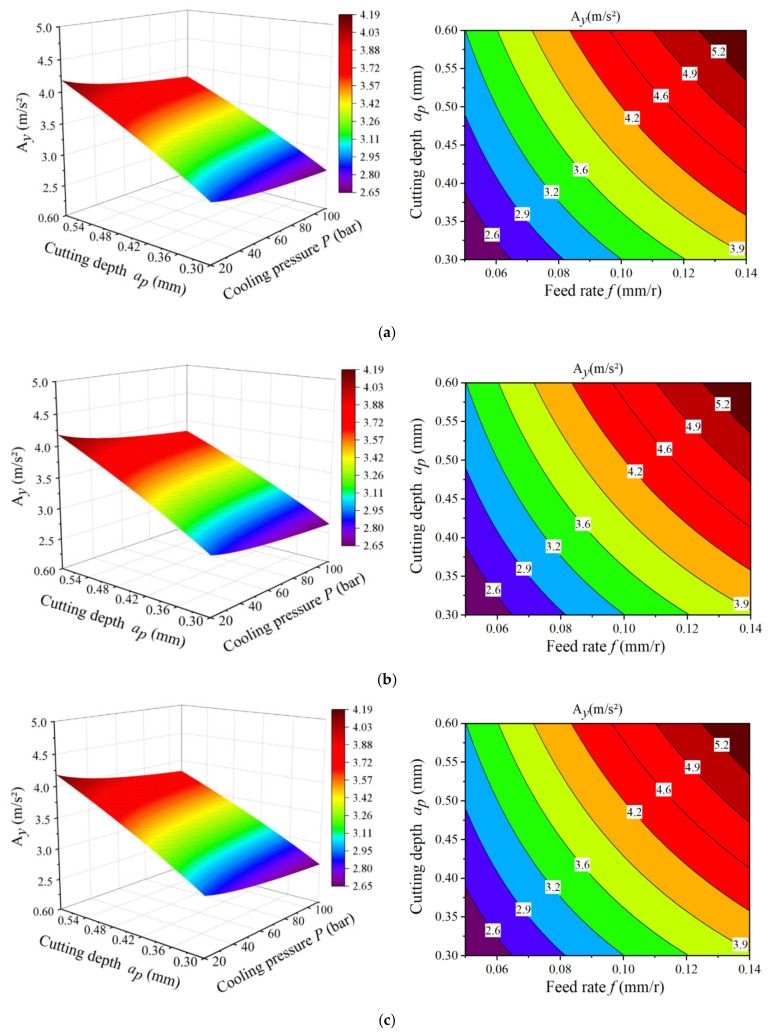

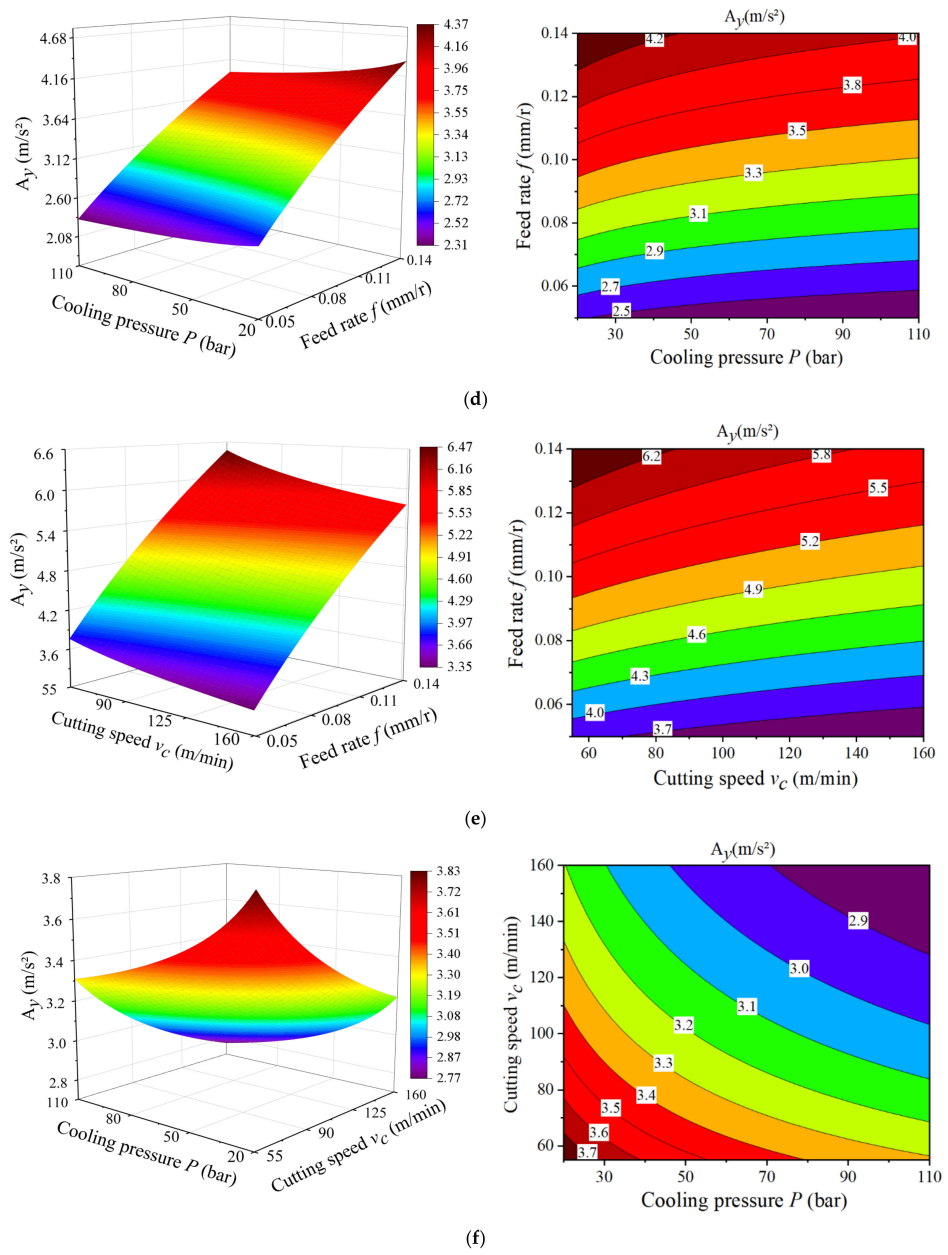
Four factors on radial tool vibration response surface diagram: (**a**) Relation of Ay with ap and P; (**b**) Relation of A_y_ with *a_p_* and *v_c_*; (**c**) Relation of A_y_ with *f* and *a_p_*; (**d**) Relation of A_y_ with *P* and *a_p_*; (**e**) Relation of A_y_ with *v_c_* and *f*; (**f**) Relation of A_y_ with *P* and *v_c_*.

This paper combined Design-Expert and Origin to solve the characteristic surface graph of any two-factor changes to visually analyze the interaction between different factors. Since the radial tool vibration acceleration was the largest and had the most obvious influence on the surface roughness, the multiple response surfaces of the radial tool vibration were established according to Table 4. Figure 13 shows the characteristic surface diagrams and contour diagrams describing the relationship between the radial tool vibration and cutting speed, feed rate, cutting depth, and cooling pressure during the cutting process of GH4169 under high-pressure cooling.

As shown in Figure 13, the interaction between the cutting depth, feed rate, cutting speed, and cooling pressure have no significant effect on the radial tool vibration. This is mainly because the high-pressure cooling pressure is constant during the cutting process. With the increase in cooling pressure, there is no sudden change in the cutting force and hence no significant increase in the tool cutting vibration. The interaction between the cutting depth, feed rate, and cutting speed had a significant impact on the radial tool vibration. The combination of a small feed rate and a low cutting depth can reduce the tool vibration. This is mainly because with the increase in the cutting depth and feed rate, the thickness and area of the cutting layer of the workpiece material increase. This causes an increase in the deformation resistance and friction during the cutting process. In addition, the frequent squeeze friction between the tool and chips increases the cutting force, thereby increasing the tool vibration acceleration. The combination of a high cutting speed and a low cooling pressure can reduce the tool vibration. This is because with the increase in the cutting speed, the tool is contacted with the workpiece and squeezed. In this case, the friction increases, which leads to an increase in the cutting temperature and causes the GH4169 workpiece material to soften during the cutting process. When the thermal softening effect of the material exceeds the plastic deformation, abrupt slip and adiabatic shearing occur in the first deformation zone, but the strain hardening in the cutting area does not occur in time. This causes a decrease in the cutting resistance and tool vibration acceleration. Subsequently, the surface roughness also decreases. However, as the cutting speed continues to increase, the thermal softening effect is weakened, and the cutting force increases, which aggravates the tool wear and then results in an increase in surface roughness.

Combining the analysis results of the single-factor experiments and the main effect diagram, it can be seen that in actual productions, to obtain the minimal tool vibration and ensure the surface quality of the machined workpiece, the feed rate should be set as small as possible; the cutting depth should be decreased as much as possible, or the parameter value of the maximum cutting depth should be limited. Under the premise of ensuring the tool life, a smaller cooling pressure should be used to increase the cutting speed.

## 4. Prediction and Optimization of Processing Parameters

### 4.1. Construction of Mathematical Model

Since the vibration acceleration trend of the three-way tool was almost the same, and the cutting amount and cooling pressure had the greatest impact on the radial tool vibration, only the radial tool vibration was considered here. The cutting speed, feed rate, cutting depth, and cooling pressure in the experiments were taken as inputs, and the radial tool vibration acceleration and surface roughness were assigned as outputs. The regress provided by the MATLAB toolbox was used to establish an exponential mathematical model between the inputs and outputs in the experiments, as shown in Formula (4):

(4) $$y_{i}=Cv_{c}^{b_{1}}f_{}^{b_{2}}a_{p}^{b_{3}}P_{}^{b_{4}}$$

where *y* is the output value; *C* is the coefficient of the mathematical model; *v_c_* is the cutting speed; *f* is the feed rate; *a_p_* is the cutting depth; *P* is the cooling pressure; and *b*_1_, *b*_2_, *b*_3_, and *b*_4_ are exponentials.

Taking logarithms on both sides of Formula (4) at the same time can be transformed into a linear expression:

(5) $$\ln(y_{i})=\ln C+b_{1}\ln(v_{c})+b_{2}\ln(f)+b_{3}\ln(a_{p})+b_{4}\ln(P).$$

Formula (5) can be represented by the following multivariate linear mathematical model:

(6) $$y=b_{0}+b_{1}x_{1}+b_{2}x_{2}+b_{3}x_{3}+b_{4}x_{4}.$$

The multiple linear regression mathematical model (6) was used to fit the surface roughness and tool vibration acceleration data collected in Table 4. The multiple linear regression mathematical model of surface roughness and radial tool vibration acceleration amplitude was obtained, which can be expressed as:

(7) $$R_{a}=-0.236+0.16x_{1}+1.18x_{2}+0.08x_{3}+0.07x_{4}$$

(8) $$A_{y}=0.218-0.158x_{1}-0.526x_{2}+0.526x_{3}-0.05x_{4}.$$

The expression of the mathematical model (4) transformed into exponential form is:

(9) $$R_{a}=0.79v_{c}^{0.16}f_{}^{1.18}a_{p}^{0.08}P_{}^{0.07}$$

(10) $$A_{y}=1.744v_{c}^{-0.158}f_{}^{0.526}a_{p}^{0.526}P_{}^{-0.05}$$

where *R_a_* denotes the surface roughness and A*_y_* denotes the amplitude of radial tool vibration acceleration.

### 4.2. Support Vector Machine Prediction

Support vector machine is an emerging machine learning algorithm developed based on the principle of structural risk minimization under the framework of statistical learning theory. This algorithm can avoid the high dependence on sample data and make the prediction values closer to the actual ones [27]. Currently, the RBF kernel function is most widely used for support vector machines. It can not only guarantee the training effect but also normalize the differentiated data. The calculation formula is shown in Equation (11):

(11) $$K\left(x_{i},x_{j}\right)=\exp⟮-\frac{{\left\|x_{i}-x_{j}\right\|}^{2}}{2\sigma^{2}}⟯$$

According to the results in Table 4, the applicable scope of the test set was determined as follows:

(12) $$\Omega =\left\{\begin{array}{c} 55\;m/\min\leq v_{c}\leq 160\;m/\min,0.3\;\mathrm{mm}\leq a_{p}\leq 0.6\;\mathrm{mm}\\ 0.03\;\mathrm{mm}/r\leq f\leq 0.14\;\mathrm{mm}/r,20\;\mathrm{bar}\leq P\leq 110\;\mathrm{bar} \end{array}\right.$$

**Figure 14:**
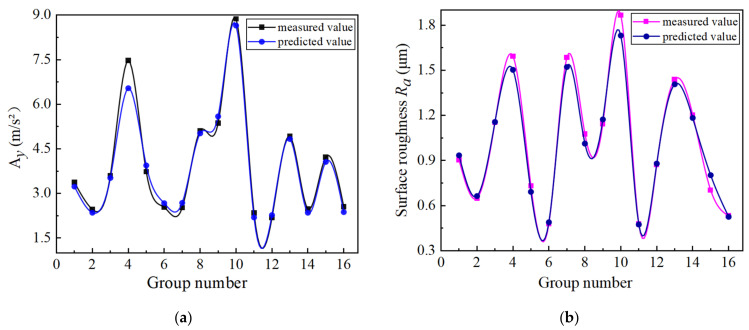
Comparison of measured and predicted values: (**a**) comparison between measured and predicted values of radial tool vibration acceleration; (**b**) comparison of measured and predicted surface roughness.

The measured values and predicted values of the output results were compared, and the comparison results are shown in Figure 14. Figure 14a,b show the comparison between the measured and predicted values of radial vibration and surface roughness, respectively.

It can be seen from Figure 14 that the measured values of radial vibration and surface roughness were consistent with the predicted values, and the error rate was almost within 10%. The small error indicates that the established mathematical models for radial vibration and surface roughness are valid, which can be used for the prediction.

### 4.3. Genetic Algorithm Optimization

**Figure 15:**
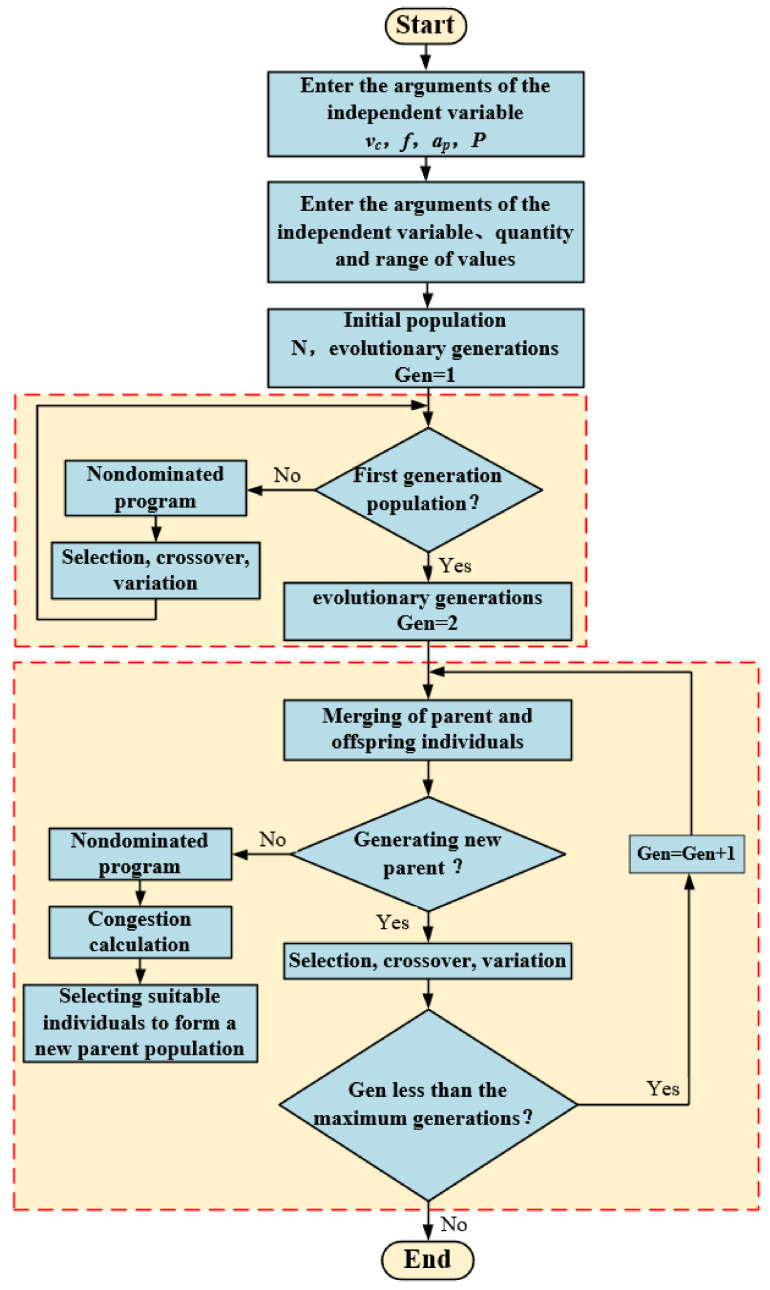
Solution flow chart of NSGA-ΙΙ.

The optimization of the cutting parameters and cooling pressure under high-pressure cooling not only aims at the surface quality and accuracy of the machining but also pursues the cutting efficiency. The NSGA-II algorithm is improved on the basis of the first generation of non-dominated sorting genetic algorithm (NSGA algorithm), which is a multi-objective optimization algorithm based on Pareto optimal solution, also known as the non-dominated sorting genetic algorithm with elite strategy, and it is one of the most popular genetic algorithms in multi-objective optimization. It has the advantages of fast running speed and good convergence of a solution set. Therefore, based on the NSGA-II algorithm, this paper comprehensively optimized the surface roughness and tool vibration amplitude by taking the cutting speed, feed rate, cutting depth, and cooling pressure as optimization variables. The calculation of NSGA-II is shown in Figure 15.

#### 4.3.1. Establishment of Multi-Objective Optimization Model

To optimize the surface roughness, radial tool vibration, and cutting efficiency, the established mathematical model for multi-objective optimization is shown in Equation (13):

(13) $$\left\{\begin{array}{c} \min R_{a}\left(v_{c},f,a_{p},P\right)\\ \min A_{y}\left(v_{c},f,a_{p},P\right)\\ \max Q\left(v_{c},f,a_{p},P\right) \end{array}\right.$$

where *R_a_* (*v_c_*, *f*, *a_p_*, *P*) and A*_y_* (*v_c_*, *f*, *a_p_*, *P*) are the mathematical models of surface roughness and radial tool vibration obtained by fitting the experimental data; *Q* (*v_c_*, *f*, *a_p_*, *P*) is the model of cutting efficiency.

According to the ranges of cutting speed, feed rate, cutting depth, and cooling pressure, the constraint conditions shown in Equations (14)–(17) were established:

(14) $$50\;m/\min\leq v_{c}\leq 160\;m/\min$$

(15) 0.03 mm/r≤f≤0.14 mm/r

(16) $$0.3\;\mathrm{mm}\leq a_{p}\leq 0.6\;\mathrm{mm}$$

(17) 20 bar≤P≤110 bar

#### 4.3.2. Optimization Calculation Based on Genetic Algorithm

**Figure 16:**
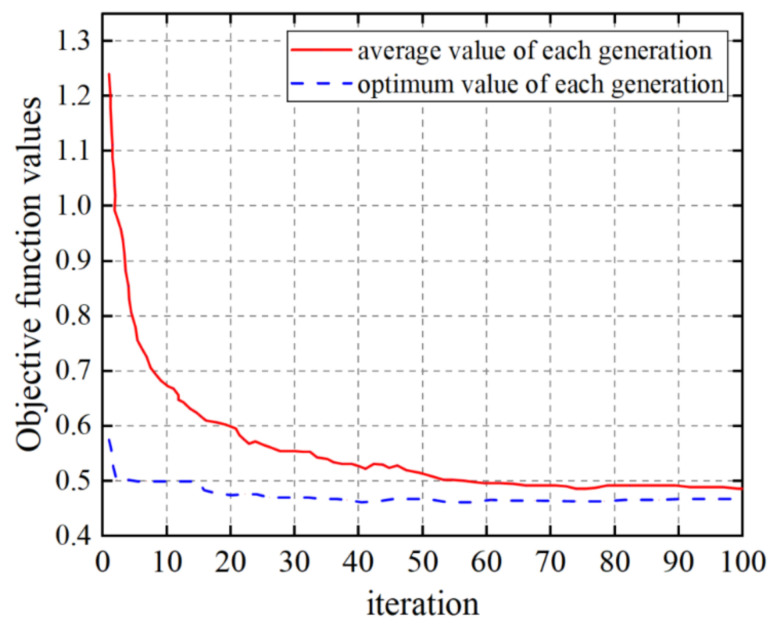
The optimization results after 100 iterations.

According to Figure 15, the above mathematical models were calculated and solved. It is supposed that the population size is 100, the maximum generation is 100, the number of iterations is 100, the crossover probability is 0.7, and the mutation probability is 0.04. The optimization process is shown in Figure 16.

It can be seen from Figure 16 that when the evolutionary generation reached 60 generations, the objective function converged. The final optimization results were as follows: the cutting speed vc was 153 m/min, the feed rate f was 0.12 mm/r, the cutting depth ap was 0.54 mm, and the cooling pressure *P* was 50 bar. At this time, the obtained radial tool vibration acceleration value and surface roughness value reached the optimal state. Meanwhile, the cutting efficiency was maximized.

#### 4.3.3. Verification of Optimization Results

To verify the validity of the optimized parameters, the optimized cutting conditions were selected for verification experiments under high-pressure cooling conditions. Table 5 lists the comparison results of the actual radial tool vibration acceleration and surface roughness values with the predicted values.

It can be seen from Table 5 that the errors between the real values and the predicted values of radial tool vibration acceleration and surface roughness were 3.79% and 5.47%, respectively. This indicates that the predicted results obtained by the optimization models are in good agreement with the experimental results. Therefore, the optimization method is reliable.

## 5. Conclusions

In this paper, regarding the processing parameter optimization of tool vibration and surface roughness, high-pressure cooling cutting experiments were conducted on the GH4169 superalloy following single-factor and orthogonal experiment methods. The main conclusions are as follows:

1. According to the single-factor experimental results and combined with the analysis of the main effect diagram, it was found that the cutting amount and cooling pressure had the same order of influence on the vibration of the three-way tools. The degree of influence in descending order was feed rate, cutting depth, cooling pressure, and cutting speed. The most significant factor affecting the surface roughness was feed rate, which was followed by the cutting speed and cutting depth and the cooling pressure.
2. The analysis of the response surface method revealed that the interaction between the cutting depth, feed rate, cutting speed, and cooling pressure had no significant effect on the radial tool vibration. The interaction between the cutting depth, feed rate, and cutting speed had a significant effect on the radial tool vibration. Especially, when the feed rate was high, the influence on the tool vibration was more obvious. In addition, the tool vibration can be reduced by the combination of a smaller feed rate and a lower cutting depth as well as a higher cutting speed and a lower cooling pressure.
3. The support vector machine was used to establish the mathematical models for radial tool vibration and surface roughness. The prediction error was within 10%, indicating that the established mathematical models can achieve accurate prediction of tool vibration and surface roughness. Meanwhile, the multi-objective genetic algorithm was also adopted for optimization. The optimization results were as follows: the cutting speed of 153 m/min, the feed rate of 0.12 mm/r, the cutting depth of 0.54 mm, and the cooling pressure of 50 bar. At this time, the obtained radial tool vibration acceleration and surface roughness values were optimal, and a higher cutting efficiency was also obtained.
4. The validity of the optimization results was verified through experiments. It was found that the errors between the experimental results and the predicted results of radial tool vibration acceleration and surface roughness were 3.79% and 5.47%, respectively. The small errors indicate that the prediction results obtained by the optimization model are consistent with the experimental results. Therefore, the selected optimization method is reliable, and it provides reference values for high-efficiency and high-quality cutting GH4169 machining under high-pressure cooling.

## Figures and Tables

**Table 1:** The main chemical composition of GH4169.

Element	Ni	Cr	Ti	Mo	Nb	Al	Co	Cu	C	Fe
Mass fraction (%)	53.38	18.40	1.1	3.00	5.13	0.38	0.076	0.022	0.017	other

**Table 2:** Parameter setting of single-factor experiment.

Test Number	Cutting Parameters	*f =* 0.05 mm/r, *a_p_ =* 0.05 mm, *P* = 50 bar				
1~5	*v_c_* (m/min)	75	100	125	150	175
**Test Number**	**Cutting Parameters**	***v_c_=* 125 m/min, *a_p_ =* 0.05 mm, *P* = 50 bar**				
6~10	*f* (mm/r)	0.03	0.05	0.07	0.09	0.11
**Test Number**	**Cutting Parameters**	***v_c_=* 125 m/min, *f =* 0.05 mm/r, *P* = 50 bar**				
11~15	*a_p_* (mm)	0.2	0.4	0.6	0.8	1.0
**Test Number**	**Cutting Parameters**	***v_c_=* 125 m/min, *f =* 0.05 mm/r, *a_p_ =* 0.05 mm**				
16~20	*P* (bar)	35	50	65	80	95

**Table 3:** Orthogonal test factor-level table.

	Factor	Cutting Speed *v_c_* (m/min)	Feed Rate *f* (mm/r)	Cutting Depth *a_p_* (mm)	Cooling Pressure *P* (bar)
Level					
1		55	0.05	0.3	20
2		90	0.08	0.4	50
3		125	0.11	0.5	80
4		160	0.14	0.6	110

**Table 4:** Orthogonal experimental results of tool vibration and surface roughness.

Experiment Number	*v_c_* (m/min)	*f* (mm/r)	*a_p_* (mm)	*P* (bar)	A*_x_* (m/s^2^)	A*_y_* (m/s^2^)	A*_z_* (m/s^2^)	*R_a_* (μm)
1	55	0.05	0.3	20	1.78	3.37	1.89	0.903
2	90	0.08	0.4	20	1.89	2.46	2.32	0.648
3	125	0.11	0.5	20	1.00	3.60	3.36	1.154
4	160	0.14	0.6	20	2.90	7.47	6.48	1.595
5	55	0.08	0.5	50	4.03	3.73	3.27	0.731
6	90	0.05	0.6	50	3.68	2.53	2.31	0.479
7	125	0.14	0.3	50	6.23	2.52	1.00	1.587
8	160	0.11	0.4	50	1.74	5.11	3.50	1.077
9	55	0.11	0.6	80	4.14	5.36	4.51	1.145
10	90	0.14	0.5	80	7.30	8.87	8.09	1.868
11	125	0.05	0.4	80	1.02	2.34	2.25	0.477
12	160	0.08	0.3	80	1.44	2.18	2.10	0.873
13	55	0.14	0.4	110	2.20	4.92	4.27	1.439
14	90	0.11	0.3	110	4.14	2.48	2.30	1.204
15	125	0.08	0.6	110	1.95	4.22	3.79	0.702
16	160	0.05	0.5	110	2.77	2.55	2.40	0.532

**Table 5:** Comparison of experimental verification results.

Radial Tool Vibration Acceleration (m/s^2^)			Surface Roughness *R_a_* (μm)		
Measured Value	Predicted Value	Error	Measured Value	Predicted Value	Error
4.22	4.06	3.79%	0.73	0.69	5.47%

## Data Availability

Not applicable.

## References

[1] Pollock T.M. (2020). Alloy design for aircraft engines. Nat. Mater..

[2] Xu J.H., Huang Z.W., Jiang L. (2017). Effect of heat treatment on low cycle fatigue of IN718 superalloy at the elevated temperatures. Mater. Sci. Eng. A.

[3] Zhang C.C., Shirzadi A.A. (2021). Diffusion bonding of copper alloy to nickel-based superalloy: Effect of heat treatment on the microstructure and mechanical properties of the joints. Sci. Technol. Weld. Join..

[4] Chu J.Y. (2016). Study on the cutting-tool selection and optimization of process parameters for high-temperature alloy materials. Master’s Thesis.

[5] Du Y.C. (2019). Dynamic Responses and Surface Topography Prediction in Micro Milling Process.

[6] Zhao T., Xiao J., Fan S., Yang F.J. (2020). Study on chip morphology and drilling force of low frequency vibration drilling of TC4 titanium alloy. China Mech. Eng..

[7] Grzesik W., Krzysztof Ż., Niesłony P., Chudy R. (2018). Detection of the occurrence of cutting vibration in surface profiles generated in CBN precision hard turning. Procedia CIRP.

[8] Zhao J.J., Pang B. (2021). Higheffciency milling parameter optimization of stringer structure parts. J. Mech. Electr. Eng..

[9] Hüseyin G., Yunus E.G. (2021). Optimization and evaluation of dry and minimum quantity lubricating methods on machinability of AISI 4140 using Taguchi design and ANOVA. Proc. Inst. Mech. Eng..

[10] Li Z.S., Shi Y.Y., Xin H.M., Zhao T., Yang C. (2018). Technological parameter optimization of disc-milling grooving of titanium alloy based on grey correlation degree. J. Northwestern Polytech. Univ..

[11] Hong-seok P., Bowen Q., Duck-Viet D., Dae Y.P. (2018). Development of smart machining system for optimizing feedrates to minimize machining time. J. Comput. Des. Eng..

[12] Kong X.Y., Yuan S.M., Zhu G.Y., Zhang W.J. (2021). Optimization of process parameters of minimum quantity lubrication system based on grey relation analysis. Aeronaut. Manuf. Technol..

[13] Wang L. (2016). Vibration Predictive Modeling and Parameter Optimization in Hardened Steel Precision Turning Process.

[14] Ratnam C., Arun V.K., Ben B.S., Murthy B.S.N. (2016). Process monitoring and effects of process parameters on responses in turn-milling operations based on SN ratio and ANOVA. Measurement.

[15] Ren X.P., Liu Z.Q., Liang X.L., Cui P.C. (2021). Effects of Machined Surface Integrity on High-Temperature Low-Cycle Fatigue Life and Process Parameters Optimization of Turning Superalloy Inconel 718. Materials.

[16] Krolczyk G.M., Maruda R.W., Nieslony P., Wieczorowski M. (2016). Surface morphology analysis of Duplex Stainless Steel (DSS) in Clean Production using the Power Spectral Density. Measurement.

[17] Podulka P. (2021). Reduction of Influence of the High-Frequency Noise on the Results of Surface Topography Measurements. Materials.

[18] Sharma A., Sheoran G., Jaffery Z.A. (2008). Improvement of signal-to-noise ratio in digital holography using wavelet transform. Opt. Lasers Eng..

[19] Pan G.Y., Zhu R. (2019). Improvement of OPAX method based on wavelet threshold denoising technique. Noise Vib. Control.

[20] Li H. (2013). Tool Vibration Analysis Based on Wavelet Packet Technique and Surface Roughness Prediction.

[21] Zhu L.D., Lian X.Q., Jiang Y.Z. (2009). Application of Wavelet transform in signal denoising and realization with MATLAB. J. Beijing Technol. Bus. Univ..

[22] Feng Y., Wang X.H. (2006). Signal De-noising Using Wavelet Transform and Realization with Matlab. J. Data Acquis. Process..

[23] Zhou P.P. (2017). Study on Cutting Vibration Characteristics of Titanium Alloy and Its Influence on Surface Roughness.

[24] Qiu K., Wang X.Y., Pang S.Q., Li B.F. (2010). Study on machinability for ironbased superalloy. J. Funct. Mater..

[25] Yuan S., He L., Zhan G., Jiang H.W., Zou Z.F. (2018). Research on surface roughness of 304 stainless steel cut by cemented carbide micro pit tool. J. Mech. Eng..

[26] Wang Y., Wang M. (2014). Research on single-factor affecting high-speed mill load with altering spindle revolution. J. Anhui Univ. Archit..

[27] Zhu X.X., Wang F.L., Jiao H.C., Han Z.H. (2017). Windspeed prediction method based on SVR and multi-parameter optimization of GA. Electr. Mach. Control.

